# Determining the influence of tree age on tea quality-related metabolites in *Camellia tachangensis* var. remotiserrata by integrating metabolomic and transcriptomic analyses

**DOI:** 10.3389/fpls.2025.1701011

**Published:** 2025-11-10

**Authors:** Zhuorong Shi, Yu Cai, Ying Tian, Yuting Zhang, Junhao Shao, Jianfeng Liu, Qingwei Zhang

**Affiliations:** 1 Key Laboratory of Eco-Environments of Three Gorges Reservoir Region, Ministry of Education, School of Life Sciences, Southwest University, Chongqing, China; 2 Chongqing Haotian Eco-Agri Tech Co., Ltd., Chongqing, China; 3 Bureau of Agriculture and Rural Affairs of Langzhong City, Nanchong, Sichuan, China

**Keywords:** black tea quality, tree age, metabolome, phenylpropanoid metabolic pathway, transcriptional factor

## Abstract

While tea tree age is generally considered to influence the quality of black tea, the relationship between tree age and tea quality is unclear. We examine relationships between tree age and key metabolite contents related to tea quality in new leaves of the wild arbor-type tea *Camellia tachangensis* var. remotiserrata. We report tree age to directly affect metabolic divergence in leaves in spring. Compared with 10-y-old trees, the leaves of 80- and 800-y-old trees have higher flavonoid contents; total soluble sugar, polyphenol, and total amino acid contents are largely unaffected by tree age, but levels of specific amino acids (e.g., theanine, glutamate, and isoleucine) decline in older trees. Metabolome analysis reveals 202 of 1041 identified compounds (primarily flavonoids, phenolic acids, organic acids, tannins, lignans, and coumarins) to exhibit significant age-dependent changes. With increased tree age, common sugar compounds (e.g., glucose, maltose) and certain lipids (e.g., 1-alpha-linolenoyl-glycerol) contents increase in leaves. Levels of relatively abundant phenolic acids (e.g., ferulic acid-4-O-glucoside, p-coumaric acid-4-O-glucoside) and flavonoids (e.g., luteolin-3’-O-glucoside, apigenin-4’-O-glucoside) decrease, while catechin content increases significantly, particularly epigallocatechin gallate derivatives. These metabolic changes align with flavor profiles of tea harvested from different tree ages: infusions from older trees are significantly less bitter and astringent and present apparent sweet and umami characteristics. Transcriptome analysis reveals overall gene expression profiles to vary with tree age, especially for genes involved in secondary metabolite biosynthesis. Focusing on the phenylpropanoid biosynthetic pathway, the expression of the genes PAL, C4H, and LAR is significantly upregulated with increased tree age, and that of flavanone 3-hydroxylase and F3GT is downregulated. Conjoint analysis of metabolome and transcriptome data reveals expression patterns of specific enzyme-encoding genes to correlate with changes in phenolic acid, flavonoid, and tannin compounds. Transcription factors (e.g., MYB, ERF, NAC) are closely associated with changes in these compounds. These results improve our understanding of relationships between tree age and tea quality. A limitation of this study is that sampling was confined to the spring season of 2023. To more definitively prove that tree age influences tea quality by altering the metabolism of young leaves, sampling across different years and a broader range of conditions is essential.

## 1 Introduction

The health benefits of tea, one of the most important beverages worldwide, are reported to include anti-diabetic, anticancer, antioxidant, anti-inflammatory, and cardiovascular-protective properties (Gan et al., 2018; Guo et al., 2017; Lin et al., 2021). Depending on processing steps, tea can be classified as black, yellow, green, dark, white, or oolong (Cai et al., 2022). Tea quality is predominantly assessed based on sensory characteristics such as aroma, flavor, and appearance. Flavor and aroma attributes depend on the diversity and concentration of constituents such as sugars, amino acids, polyphenols, flavonoids, and alkaloids (Chaturvedula and Prakash, 2011; Wang et al., 2022; Zhang et al., 2020) that are influenced by factors such as tea variety, cultivation environment (climate, soil, altitude), management practices, harvest timing, and processing techniques (Feng et al., 2019; Huang et al., 2018; Jahan et al., 2022; Liu et al., 2021; Xu et al., 2022). The age of the tree from which leaves are collected is also regarded to be important, but is seldom researched.

Age influences morphological, physiological, and molecular processes throughout a plant’s life cycle. Age-dependent changes affect resource allocation, hormone regulation, gene expression, enzymatic activity, and metabolic efficiency that collectively determine plant resilience, productivity, and survival. As tea trees age, metabolic pathways of various compounds are modified in response to the environment, and these affect tea quality—partly explaining why teas sourced from older trees are highly valued in many tea-producing regions. However, how tree age affects taste- and aroma-related substances is poorly understood.

The tea plant Nanchuan Dachashu (commonly, formerly known as *Camelia nanchuanica*) was recently reclassified as *C. tachangensis* var. remotiserrata (Shen et al., 2025). As an arbor-type tea plant, its wild population occurs mostly on Jinfo Mountain (Chongqing, China)—a distribution that provides unique conditions to investigate how tree age affects metabolic pathways. We examine young leaves (one bud and two leaves) from trees of distinct ages, on which we perform metabolome and transcriptome analyses. Contents of total sugars, polyphenols, flavonoids, and amino acids from these leaves are reported to better understand the influence of tree age on tea quality.

## 2 Materials and methods

### 2.1 Materials

In Delong Village (28°47′N, 107°01′E), Nanchuan District, Chongqing, a tea plant population under the care of a farming family was selected as the research subject. This population includes three wild tea trees of approximately 800 years old—the oldest specimens—growing in close proximity, along with tea plants propagated from cuttings of these wild trees, aged 80 and 10 years, respectively. These three approximately 800-year-old wild tea trees have been officially identified and designated as ancient and famous trees by the Chongqing Municipal Commission of Agriculture and Rural Affairs and the Chongqing Forestry Administration (Wang et al., 2003), with identification numbers 019, 020, and 021. The growth status of tea trees see **Supplementary Figure S1** and their growth characteristics are detailed in **Supplementary Table S1**. The statistical analysis revealed significant differences in diameter at breast height, tree height, and crown width among tea plants of different ages, thus confirming substantial age variation among the sampled specimens. Trees grow naturally and are fertilized annually (autumn) with composted cow manure. Tea shoots, harvested on April 1, 2023, comprised two leaves with an apical bud; shoots were flash-frozen in liquid nitrogen, and stored at −78°C.Each treatment had 15 shoots,and three independent biological replicates were established during sampling.

### 2.2 Widely-targeted metabolomic analysis

Widely-targeted metabolite identification and quantification were performed by MetWare Company (Wuhan, China) (https://www.metware.cn/). Leaf samples were freeze-dried and homogenized by grinder (MM 500 VARIO, Retsch, Germany) at 30 Hz for 150 s. Then, 100 mg powder was extracted with 1 mL of 75% methanol containing 0.1 mg·L^−1^ lidocaine (internal standard) at 5°C for 12 h. After centrifugation (12,000 × g, 15 min, 5°C), supernatants were filtered through 0.22 µm membranes (Millipore Corp., UK). Metabolomic analysis was performed on a UPLC-ESI-MS/MS system (Applied Biosystems 4500 QTRAP, Thermo Fisher Scientific, Waltham, USA). The UPLC conditions were as follows: 1) Column, Agilent SB-C18 (1.8 μm, 2.1 mm × 100 mm); 2)Mobile phase, phase A consisted of ultrapure water (with 0.1% formic acid), and phase B was acetonitrile (with 0.1% formic acid); 3) Elution gradient, 5% B at 0.00 min, increased linearly to 95% B over 9.00 min, maintained at 95% B for 1 min, then decreased to 5% B from 10.00 to 11.10 min, and finally re-equilibrated at 5% B until 14.00 min; 4) Flow rate, 0.35 mL/min; column temperature set at 40°C, injection volume set as 4 μL. Electrospray ionization parameters included: source temperature, 550°C; ion spray voltage, +5500 V and −4500 V; gas pressures (psi): ion source gas I (50), gas II (60), curtain gas (25); collision-activated dissociation: high instrument calibration used 10 μmol L^−1^ (QQQ mode) and 100 polypropylene glycol (LIT mode) solutions. QQQ scans used MRM experiments with medium collision gas (N_2_). Declustering potential and collision energy were optimized for individual MRM transitions, with specific transitions monitored per elution period. Metabolites were identified/quantified by comparing retention times, m/z values, and fragmentation patterns with authentic standards or via database matching (KNApSAcK, MassBank, METLIN, MoTo DB). For quality control, triplicate samples were prepared by pooling 15 g of powder from each sample.

### 2.3 RNA-sequencing

Total RNA was extracted from each sample using TRIzol reagent (Invitrogen, NJ, USA) following manufacturer protocols. RNA integrity was verified by 1% agarose gel electrophoresis, and concentration was measured using a Qubit 2.0 Fluorometer (Life Technologies, CA, USA). For RNA sequencing, 1 μg of RNA per sample was processed: mRNA was purified from total RNA with poly-T oligo-attached magnetic beads, fragmented using divalent cations, and reverse-transcribed into first-strand cDNA using random hexamer primers. Second-strand cDNA synthesis was performed with DNA Polymerase I and RNase H. Resulting cDNA was purified using the AMPure XP system (Beckman Coulter, Beverly, USA). Size-selected, adapter-ligated cDNA was treated with 3 μL USER Enzyme (NEB, USA) at 37°C for 20 min, followed by 95°C incubation for 6 min prior to PCR amplification. PCR was performed with Phusion High-Fidelity DNA polymerase, Universal PCR primers, and Index (X) Primer. PCR products were purified with the AMPure XP system, and library quality was assessed on an Agilent Bioanalyzer 2100 system.

Raw reads were filtered to remove adapters and ambiguous sequences, yielding clean reads. These were aligned to the *C. sinensis* var. assamica yinghong9 reference genome (https://www.tea-pangenome.cn/, provided by Chen et al., 2023) using HISAT2 v2.1.0. The number of clean reads and gene mapping rate see **Supplementary Table S2**. Gene expression levels were quantified as FPKM (Fragments Per Kilobase of exon per Million mapped fragments) with featureCounts v1.6.4 and StringTie v1.3.4. Differential expression analysis was performed using DESeq2, with transcripts satisfying FDR < 0.05 and |log_2_(fold change)| ≥ 1 classified as differentially expressed genes (DEGs). Metabolic pathways and gene functions were annotated using the Kyoto Encyclopedia of Genes and Genomes (KEGG) database.

### 2.4 Amino acid contents

Samples were ground into powder under liquid nitrogen. Aliquots (0.1 g) were transferred to tubes and mixed with 10 mL of ultrapure water. After vortexing, tubes were heated in a 100 °C water bath for 30 min. Mixtures were centrifuged at 10,000 g for 15 min. Then, 200 μL of supernatant was combined with 200 μL 2,4-dinitrofluorobenzene solution, 200 μL Na_2_CO_3_-NaHCO_3_ buffer (pH 9.16), and 200 μL ultrapure water in a 2 mL centrifuge tube. The mixture was incubated in a 60°C water bath for 1 h in darkness. After cooling to room temperature, 800 μL KH_2_PO_4_-NaOH buffer (pH 7.0) was added, vortexed for 1 min, and kept in darkness for 15 min. Solutions were then filtered through 0.22 μm aqueous membranes for Liquid Chromatography (LC) analysis, with system [Thermo Ultimate 3000; Column, YMC-Pack ODS-AQ AQ12S05-1546WT (5 μm, 150 × 4.6 mm)] properties: mobile phase A, 4 mmol L^−1^ sodium acetate/tetrahydrofuran (96:4, v/v), and B, 80% acetonitrile (v/v); detection, 360 nm; temperature, 35°C (column/detector); injection volume, 10 μL; flow rate, 0.9 mL min^−1^; and gradient program: 0–30 min (2% → 27% B), 30–40 min (27% → 50% B), and 40–50 min (50% → 2% B).

### 2.5 Gene expression

Quantitative reverse transcription polymerase chain reaction (qRT-PCR) was performed to analyze candidate gene expression using Ubiquitin as the reference gene. The selected genes and corresponding primers see **Supplementary Table S3**. cDNA was synthesized using the PrimeScript RT Master Mix kit (Takara, RR036A). qRT-PCR reactions (15 μL total volume) contained 7.5 μL Evagreen dye (Biotium), 1.5 μL cDNA, 60 nM primers, and nuclease-free water. Reactions were run in triplicate on 96-well plates (Bio-Rad) at 95°C for 10 min, and 40 cycles of 95°C for 15 s and 60°C for 1 min. Melting curve analysis confirmed amplification specificity. Threshold cycle values were determined from three biological replicates with three technical replicates each.

### 2.6 Total soluble sugar content

Fresh leaves (0.2 g) were homogenized in 1 mL ultrapure water and transferred to a 25 mL tube. The mortar was rinsed four times with 4 mL ultrapure water (16 mL total rinse volume). After adding 15 mL ultrapure water, samples were incubated at 90°C for 1 h. Extracts (3 mL) were centrifuged (5000 g, 15 min, 4°C), and supernatants were stored at 4°C in darkness. For the standard curve, sucrose solutions (0–100 μg mL^−1^) were mixed with 0.5 mL anthrone (0.02 g mL^−1^) and 5 mL sulfuric acid, incubated at 100°C for 10 min, and measured at 620 nm. Tea extracts were analyzed identically. Total sugar content was expressed as mg g^−1^ fresh weight (FW).

### 2.7 Total polyphenol content

Liquid nitrogen-ground powder (0.1 g) was extracted in 10 mL 80% methanol at 50°C for 60 min. After centrifugation (12,000 rpm, 15 min), supernatants were collected. Standard gallic acid solutions (0–40 μg mL^−1^) were reacted with 7 mL 1 mol L^−1^ H_2_SO_4_ and 2 mL 0.328 mg mL^−1^ KMnO_4_ at 55°C for 15 min. Absorbance at 525 nm was measured after cooling. Tea extracts were processed identically. Total polyphenols are expressed as mg g^−1^ FW.

### 2.8 Total flavonoid contents

Powdered samples (0.15 g) were sonicated (3500 Hz, 30 min) in 3 mL 70% methanol at room temperature (3 cycles, 2 h intervals). After centrifugation (4000 rpm, 15 min, 4°C), 1 mL supernatant was diluted to 20 mL with 70% methanol, filtered (0.22 μm), and stored at 4°C. Rutin standards (10–50 μg mL^−1^) were analyzed via LC (Xtimate C18 column, 4.6 × 250 mm, 5 μm; mobile phase A, acetonitrile, and B, 0.1% acetic acid [7:3 v/v]; flow rate: 0.9 mL min^−1^; 40°C; injection, 10 μL). Tea extracts were analyzed identically. Total flavonoid content is expressed as mg g^−1^ FW.

### 2.9 Statistical analysis

Data are presented as mean ± SEM. Analyses were conducted using GraphPad Prism 9. Two-group comparisons used Student’s t-test (two-tailed); multi-group comparisons used one-way ANOVA with Tukey’s *post-hoc* test. Significance was defined at p < 0.05.

## 3 Results

### 3.1 Effect of tree age on soluble sugars, polyphenols, flavonoids, and amino acids in leaves

**Figure 1:**
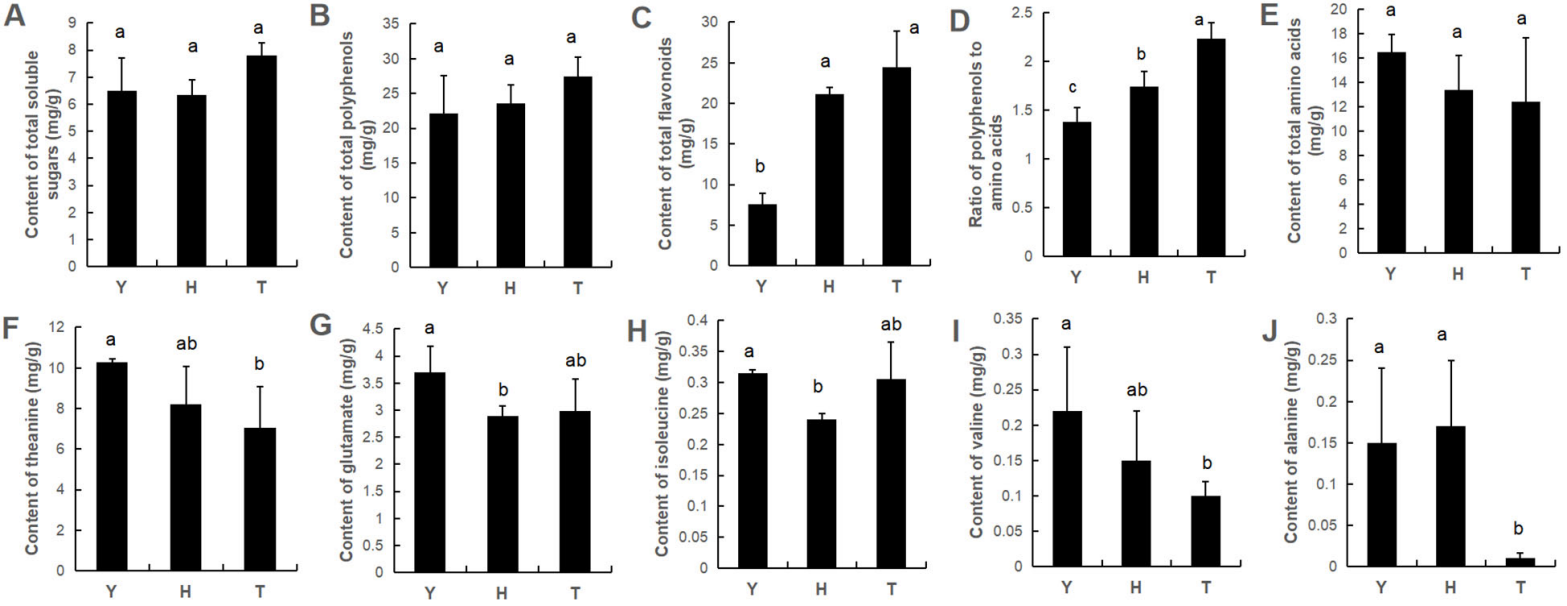
The content of total soluble sugars **(A)**, total polyphenols **(B)**, total flavonoids **(C)**, ratio of polyphenols to amino acids **(D)** and total amino acids **(E)**, as well as the content of specific amino acid **(F–J)** in young leaves of tea plants with various ages. Y: 10-year old; H: 80-year old; T: 800-year old. Different lowercase letters (a, b, c) indicate a significant difference (ANOVA, P < 0.05).

Tea flavor is closely associated with flavor-contributing components such as soluble sugars, polyphenols, amino acids, and flavonoids (flavonols). No significant differences occurred in total contents of soluble sugars, polyphenols and amino acids in leaves of tea trees of different ages (**Figure 1**). However, total flavonoid content tended to increase with tree age, and the phenol–amino acid ratio also increased with tree age. In terms of specific amino acids, we found levels of specific amino acids to be affected by tree age—specifically, theanine, glutamic acid, valine, and alanine—the levels of which were significantly higher in leaves of 10-y-old tea trees than in 80- and 800-y-old tea trees (**Figure 1**).

### 3.2 Effect of tree age on tea leaf metabolism

**Figure 2:**
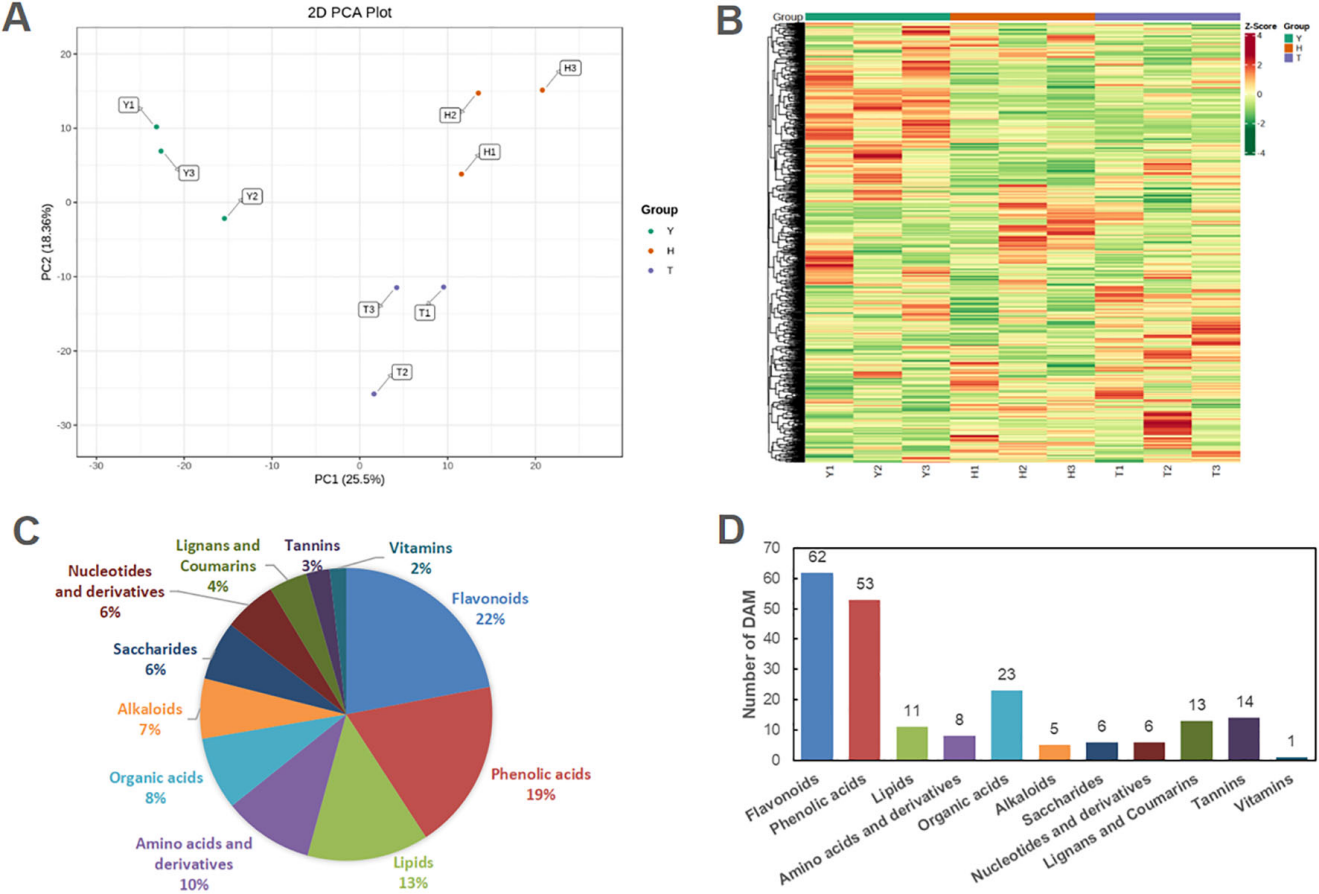
The overall metabolic divergence in tea plants with various ages. **(A)** The PCA scatter plot of samples based on metabolomic dataset. **(B)** The heatmap of all the identified metabolites from different samples. **(C)** Classification of all the identified metabolites in young leaves of tea plant. **(D)** The number of differentially accumulated metabolites (DAMs) in various paired-comparisons with the criteria of Log2 |(Fold change)| ≥ 1 and VIP values ≥ 1.0. Y: 10-year old. H: 80-year old. T: 800-year old.

To better understand the influence of tree age on leaf metabolism and quality components, metabolome analysis was performed. Of 1041 identified compounds, PCA revealed three clearly separated groups of samples, indicating significant changes in the metabolites of leaves from trees of different age (**Figure 2**). Heatmap analysis also revealed significant differences in the metabolite composition of leaves from tea trees of different age (**Figure 2**). Among identified compounds, there were 69 alkaloids, 104 amino acids and derivatives, 229 flavonoids (including flavones, flavonols, and flavanols), 44 lignins and coumarins, 140 lipids, 61 nucleosides (nucleotides) and derivatives, 84 organic acids, 68 saccharides, 19 vitamins, 196 phenolic acids, and 27 tannins (including proanthocyanidins). Of these, 202 compounds showed significant differences among trees of different ages (**Figure 2**), with flavonoids, phenolic acids, and organic acids being the most numerous.

**Figure 3:**
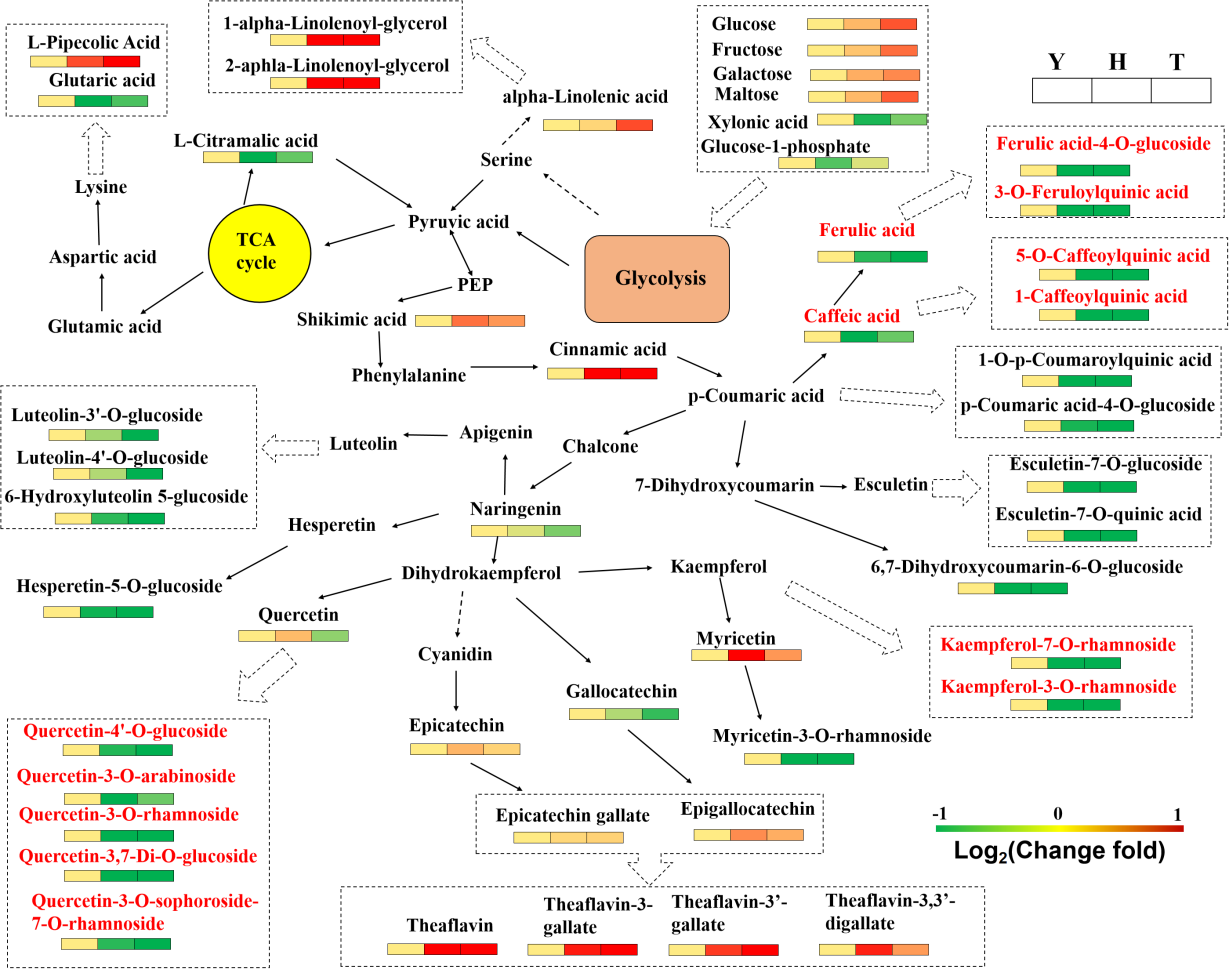
Network diagram of metabolic pathways influenced by tree age. The heatmap was plotted using the relative change fold; the cells, from left to right, represented Y(10-year old), H(80-year old) and T (800-year old), respectively. TCA cycle, tricarboxylic acid cycle.

In summary, with increased tree age, the contents of common sugars (glucose, fructose, galactose, and maltose) in leaves increased (**Figure 3**), whereas levels of xylonic acid and glucose 1-phosphate decreased. Of lipids, the most typical change involved an increase in contents of 1-alpha-linolenoyl-glycerol and 2-aphla-linolenoyl-glycerol (which occurred dozens of times). Contents of several organic acids were also higher in leaves of 10-y-old tea trees than in those of 80- and 800-y-old trees (e.g., glutaric, trans-citridic, L-citramalic, and 4-acetamidobutyric acids).

Of secondary metabolites, we report the contents of most phenolic acid substances (e.g., ferulic acid-4-O-glucoside, p-coumaric acid-4-O-glucoside, 1-O-p-coumaroylquinic acid, 3-O-feruloylquinic acid, and 5-O-caffeoylquinic acid) to tend to decrease in leaves of increased tree age (**Figure 3**). Some phenolic acids (e.g., caffeic acid and 3-O-galloyl-D-glucose) have lower levels in leaves of 80-y-old trees than in 10- and 800-y-old trees. Phenolic acids, along with catechins and flavonol/flavone glycosides, are involved in the flavonoid metabolic pathway. Different trends suggest that tree age differentially regulates the downstream branches of the flavonoid metabolic pathway. We report the main flavonols with relatively high content in leaves to be kaempferol glycosides, quercetin glycosides, and myricetin glycosides (**Figure 3**). Contents of most of these substances decrease with increased tree age. Flavones with higher contents in leaves include luteolin-3’-O-glucoside, apigenin-4’-O-glucoside, and hesperetin-5-O-glucoside. Luteolin and hesperetin contents decrease with tree age, and apigenin increases.

Tannins, lignans, coumarins, and catechins were affected by tree age. Contents of many tannin compounds (e.g., gemin D, isostrictinin, isocorilagin, strictinin) were significantly lower in leaves of 80-y-old trees than in 10- and 800-y-old trees (**Supplementary Figure S2**). In contrast, some tannin compounds related to tea color (e.g., theaflavin and theaflavate B) had higher contents in leaves of 80- and 800-y-old trees. Contents of lignans and coumarins (e.g., 3,4-methylenedioxy cinnamyl alcohol, esculetin-7-O-glucoside, esculetin-7-O-quinic acid, and 6,7-dihydroxycoumarin-6-O-glucoside) mainly trend down with increased tree age (**Supplementary Figure S1**). Catechins are considered to be the most important flavonoid substances in tea leaves, with epicatechin being the predominant one. Under the action of a series of enzymes, they can polymerize to form proanthocyanidins, theasinensins, and other dimeric catechin substances. There were no significant differences in contents of major catechins (e.g., epigallocatechin (EGC), gallocatechin gallate (GCG), and EGCG) in leaves of trees of different ages (**Figure 3**). Of dimeric catechins, the contents of theaflavin and theaflavin-3-gallate in leaves of 80- and 800-y-old trees were significantly higher than in 10-y-old trees. Metabolomics (the increase in the content of catechins and their dimers) was consistent with that of total polyphenol content determination in leaves.

Additionally, significant variations in content were observed for only four alkaloids and three nucleotides (including their derivatives) across the tea plant samples of different ages (**Supplementary Figure S3**). Of these compounds, methyl nicotinate and adenosine demonstrated the most marked alterations. Specifically, the concentration of methyl nicotinate was higher in younger tea plants, whereas the level of adenosine showed a positive correlation with increasing age.

### 3.3 Effect of tree age on gene expression in leaves

**Figure 4:**
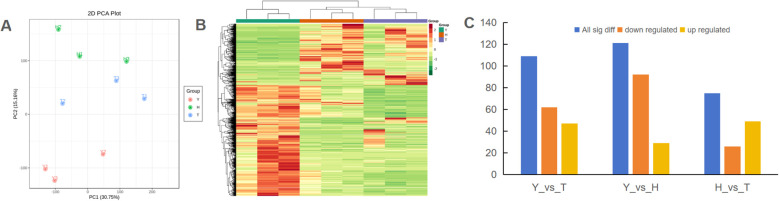
The overall gene expression divergence in tea plants with various ages. **(A)** The PCA score plot of different samples based on the transcriptomic dataset. **(B)** The heatmap of all identified genes in different samples. **(C)** the numbers of differentially expressed genes (DEGs) in various paired-comparisons with the criteria of Log2 |(Fold change)| ≥ 1 and false discovery rate < 0.05. Y: 10-year old H: 80-year old; T: 800-year old.

To determine the impact of tree age on overall gene expression, we performed transcriptome analysis. A total of 516,424,304 raw reads were obtained (**Supplementary Table S2**). After removing sequencing adapter contamination and low-quality sequences, 498,188,438 high-quality clean reads were generated, totaling 74.74 G of clean bases. The Q20 of clean reads was > 97.0%, Q30 was > 91.9%, and the overall sequencing error rate was 0.03%, indicating that sequencing data were of good quality and suitable for subsequent analysis. Screening criteria for DEGs were set as |log2 fold change| ≥ 1 and FDR < 0.05. Compared with 10-y-old trees, 80-y-old trees produced 1,055 DEGs, of which 605 were upregulated. Compared with 10-y-old trees, 800-y-old trees yielded 975 DEGs, of which 733 were upregulated (**Figure 4**). Compared with 80-y-old trees, the 800-y-old trees produced 174 DEGs, including 101 that were upregulated. For validation of the transcriptome sequencing results, qRT-PCR was performed on 33 genes with functions related to carbohydrate metabolism, lipid metabolism, amino acid metabolism, secondary metabolism, plant hormone response, cell division, and cell wall formation (**Supplementary Figure S4**). The analysis revealed a correlation coefficient (R) of >0.8 between the expression levels and FPKM values for 24 genes, and >0.6 for 30 genes. A negative correlation (R = -0.304) was observed for only one gene. These findings confirm the reliability of the transcriptomic analysis, supporting its use for subsequent investigations. Overall gene expression heatmap and PCA results for gene expression reveal the gene-expression trends of samples from 80-y-old trees to be relatively consistent with those of 800-y-old trees (**Figure 4**).

### 3.4 KEGG Functional enrichment analysis of DEGs

**Figure 5:**
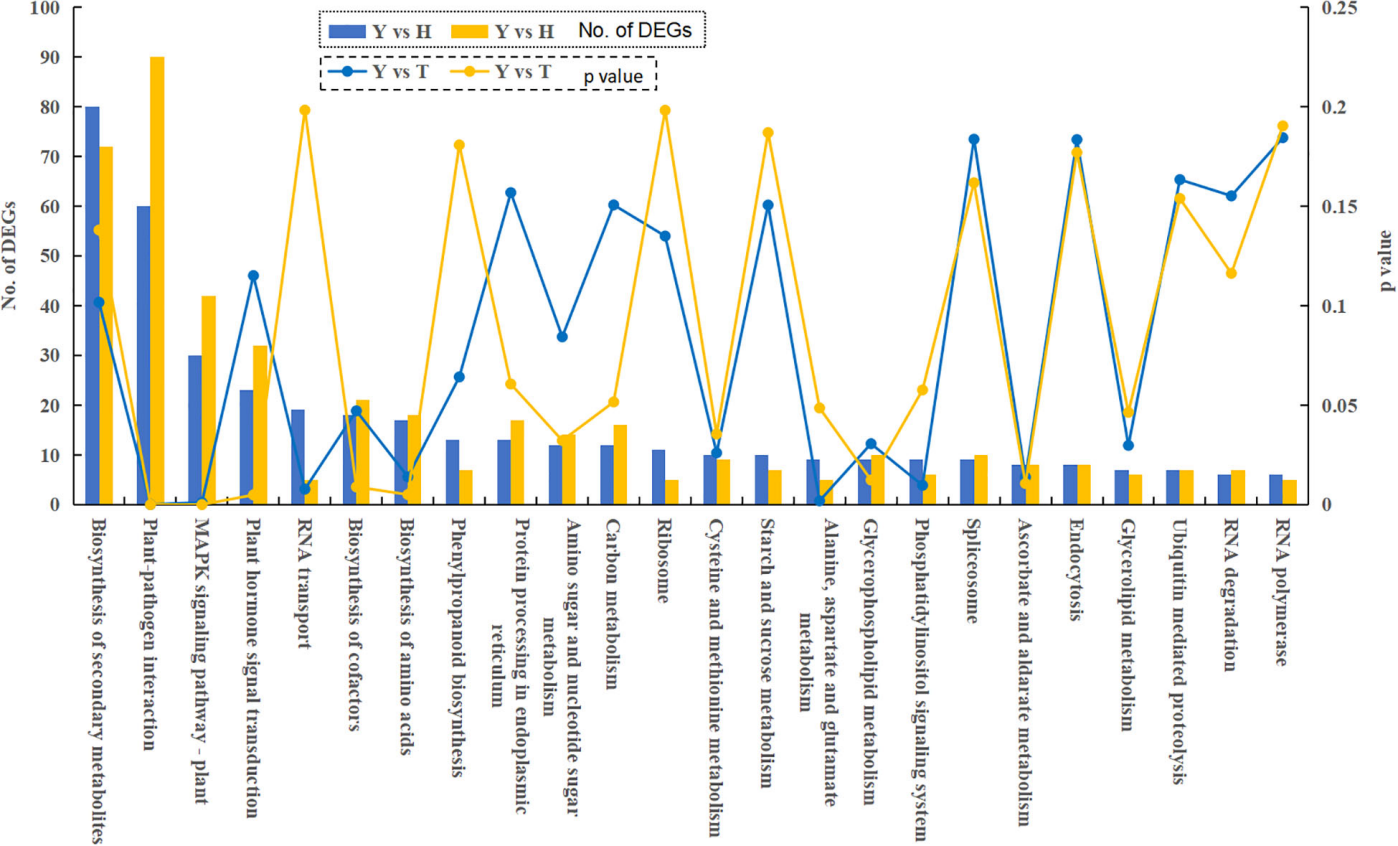
KEGG enrichment analysis of differentially expressed genes in the paired comparisons of H/Y and T/Y. Y: 10-year old; H: 80-year old; T: 800-year old.

KEGG functional enrichment analysis was performed on DEGs from trees of 10, 80, and 800 y age, resulting in a total of 91 enriched metabolic pathways. Analysis of the top 25 pathways revealed most DEGs to be involved in multiple metabolic processes related to amino acid, nucleotide, lipid, carbohydrate, and secondary metabolism (**Figure 5**). Pathways annotated by these DEGs are concentrated in primary and secondary metabolites and related signaling pathways, suggesting that the aging process of tea plants modulates metabolic pathways through alterations in gene expression profiles.

### 3.5 Expression of genes related to the phenylpropanoid metabolic pathway

**Figure 6:**
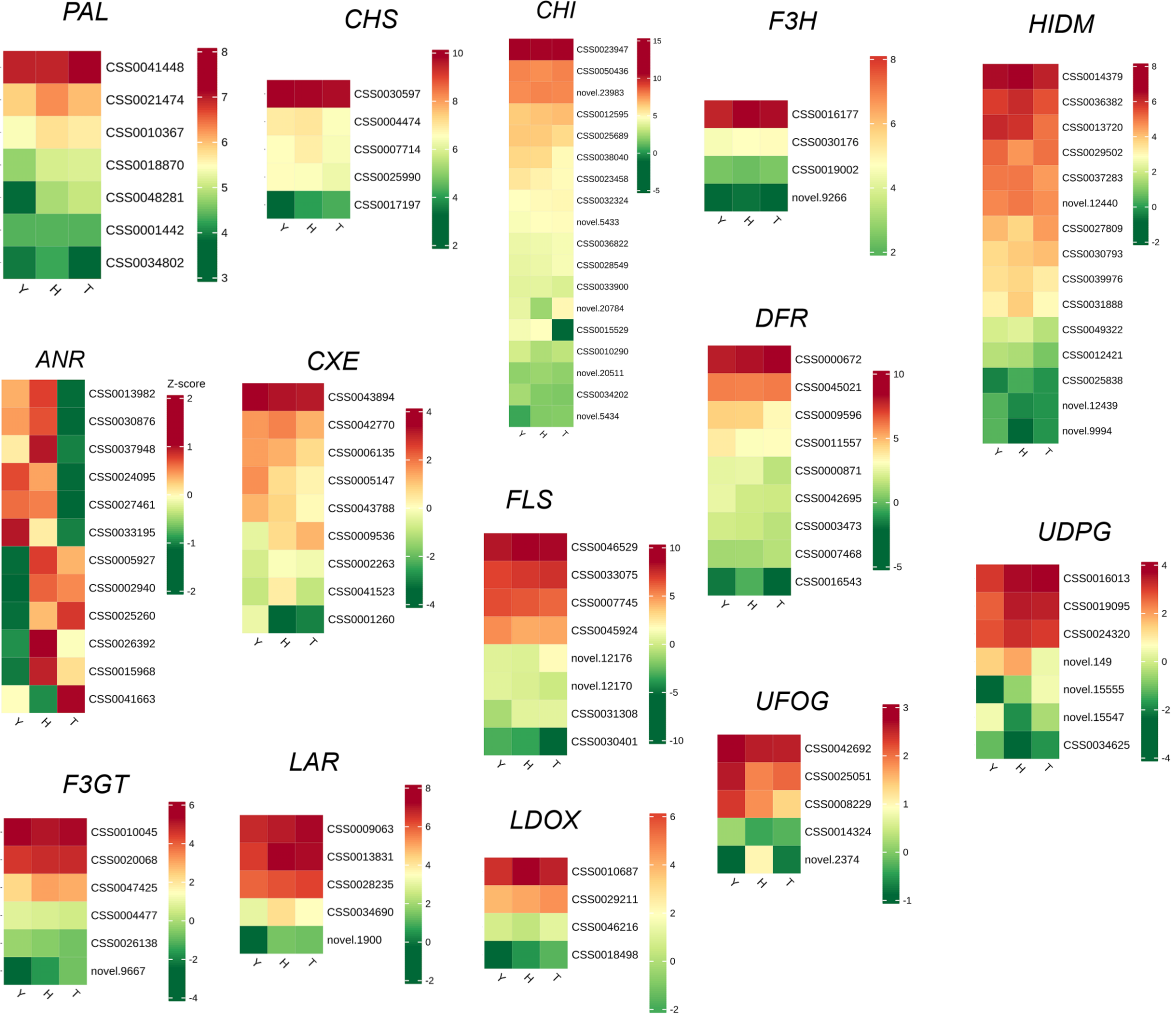
The expression heatmap of genes involving phenylpropanoid metabolic pathway. Y: 10-year old; H: 80-year old; T: 800-year old. PAL, Phenylalanine ammonia-lyase; CHI, Chalcone isomerase; CXE, carboxylesterase; DFR, Dihydroflavonol 4-reductase; F3H, Flavonoid 3’-monooxygenase; FLS, Flavonol synthase/flavanone 3-hydroxylase; HIDM, 2-hydroxyisoflavanone dehydratase; ANR, anthocyanidin reductase; F3GGT, Flavonoid 3-O-glycoside glycosyltransferase; FAOMT, Anthocyanin-O-methyltransferase; LAR, Leucoanthocyanidin reductase; LDOX, Leucoanthocyanidin dioxygenase; UFOG, UDP-glucose flavonoid 3-O-glucosyltransferase 3.

Both metabolomics and transcriptomics studies demonstrate tree age to significantly affect levels of phenolic acids, flavonoids, lignans, coumarins, and tannins in tea leaves. These substances are closely associated with the phenylpropanoid metabolic pathway in plant cells. Specifically, the key enzyme chalcone synthase catalyzes the condensation of p-coumaroyl-CoA with three molecules of malonyl-CoA to form chalcone. Chalcone is then converted to flavanone by chalcone isomerase, which serves as the precursor for other flavonoid classes. Downstream enzymes such as flavanone 3-hydroxylase, dihydroflavonol 4-reductase, and anthocyanidin synthase further modify the flavanone backbone to produce diverse flavonoid compounds, including flavonols, flavones, anthocyanidins, and proanthocyanidins. Additional modifications such as glycosylation, methylation, and acylation contribute to flavonoid structural diversity (Dong and Lin, 2021). Therefore, particular attention was devoted to genes closely associated with the phenylpropanoid metabolic pathway (chalcone synthase, chalcone isomerase, flavanone 3-hydroxylase, dihydroflavonol 4-reductase, and anthocyanidin synthase, as well as enzymes involved in glycosylation, methylation, and acylation reactions) (**Figure 6**). We report the expression of many genes involved in the synthesis of phenolic acids and flavonoids to differ strongly among trees of different age, especially ANR, which differs completely between trees of 10 and 80 and 800 y age, possibly explaining differences in flavonoid and tannin levels between them. To verify the influence of tree age on phenylpropanoid metabolic pathway, qPCR analysis was performed (**Supplementary Figure S5**). With increased tree age, the intensity of secondary metabolism in leaves, especially the phenylpropanoid metabolic pathway, was enhanced—evidenced by the significant upregulation of PAL, chalcone isomerase, C4H, and LAR gene expression levels. The impact of tree age on phenylpropanoid metabolic pathways is direction-specific, manifested as a progressive attenuation in flavonoid synthesis with increased tree age (accompanied by decreases in expression levels of genes flavanone 3-hydroxylase, and F3GT). 3.6. Integrated metabolomics and transcriptomics analyses.

**Figure 7:**
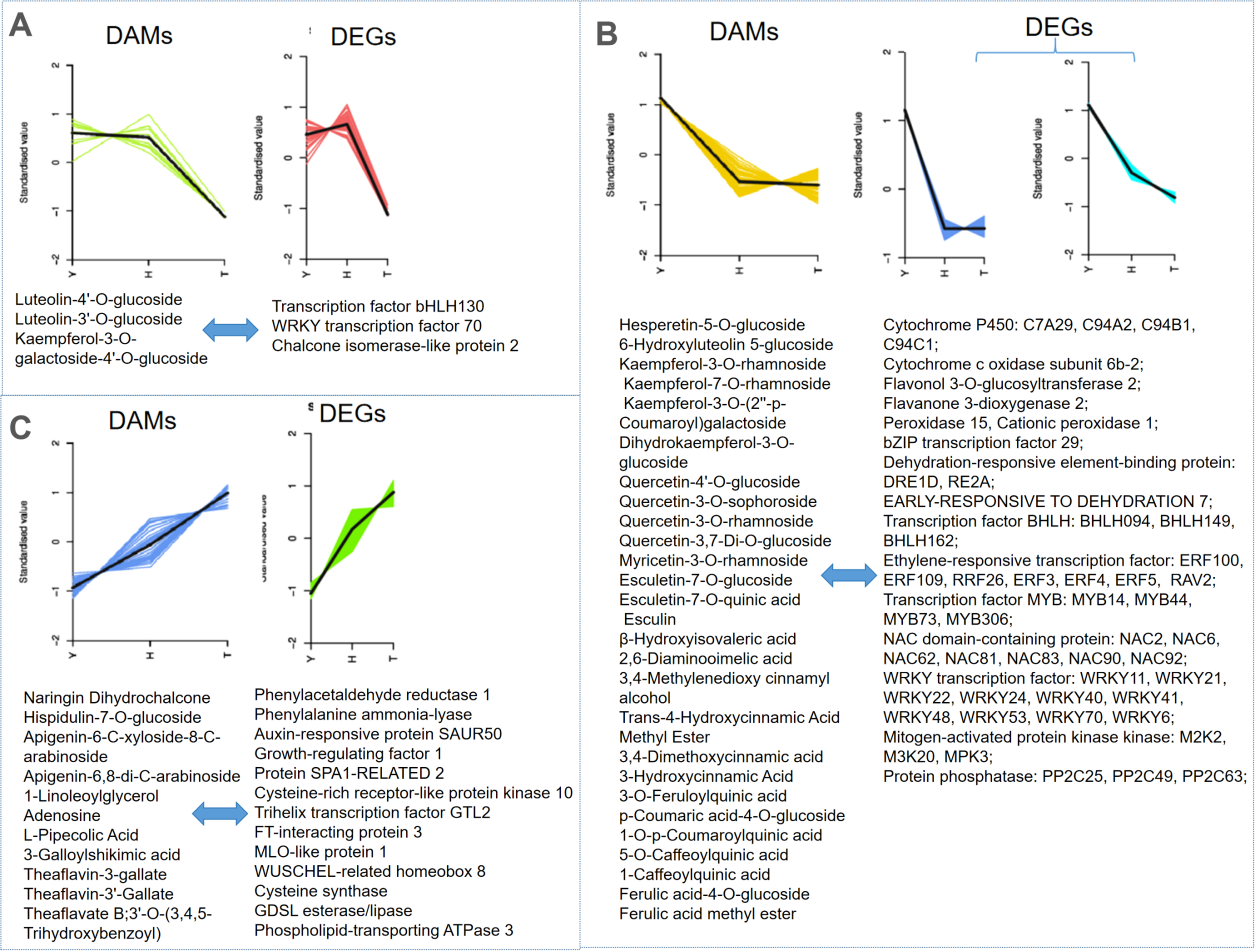
The differentially accumulated metabolites (DAMs) and differentially expressed genes (DEGs) with concordant trends and strong correlation. Y: 10-year old; H: 80-year old; T: 800-year old. **(A–C)** indicate different kinds of trends.

To better understand relationships between gene expression and metabolite levels, a cluster analysis was performed on both differentially accumulated metabolites (DAMs) and DEGs (**Supplementary Figure S6**). Because we sought to investigate the impact of tree age on metabolism and gene expression in leaves, we focused on DAMs and gene clusters that positively and negatively correlated with tree age. Specifically, we analyzed subclasses 3, 4, and 6 for the former, and 6 and 8–10 for the latter. Additionally, we performed correlation analysis between DAMs and DEGs (**Figure 7**).

With increased tree age, relatively few compounds exhibited a “high–high–low” pattern, and those that did were mainly phenolic acids and flavonoids such as luteolin-4’-O-glucoside and kaempferol-3-O-galactoside-4’-O-glucoside (**Figure 7A**). Genes for which expression patterns were consistent with changes in these compounds included transcription factors (TFs) bHLH130 and WRKY 70.

With increased tree age, many compounds showed a “high–low(mid)–low” pattern, with those that did mainly including flavonoids and phenolic acids (e.g., hesperetin-5-O-glucoside, esculetin-7-O-glucoside, 3-O-feruloylquinic acid, and 2,6-diaminooimelic acid) (**Figure 7B**). Genes for which expression patterns were consistent with changes in these compounds included key enzymes in the phenylpropanoid metabolic pathway (e.g., flavanone 3-dioxygenase 2 and peroxidase) and numerous and various TFs (involving MYB, ERF, NAC, WRKY, MAPK, and PP2C). qPCR analysis revealed gene expression levels of MYC2, ERF3, and WRKY40 to trend downwards with increased tree age, and expression levels of MYB86 and MYB123 to be higher in 80- and 800-y-old tea trees than in 10-y-old tea trees (**Supplementary Figure S5**).

Compounds with a “low–mid–high” pattern with increased tree age were mostly flavonoids and tannins, with a few organic acids and lipids such as naringin dihydrochalcone, 1-linoleoylglycerol, adenosine, L-pipecolic acid, and theaflavin-3’-gallate (**Figure 7C**). Genes with expression patterns consistent with changes in these compounds included some related to plant secondary metabolism (e.g., phenylalanine ammonia-lyase and GDSL esterase/lipase) and several TFs (auxin-responsive protein SAUR50, growth-regulating factor 1, SPA1-RELATED 2, and cysteine-rich receptor-like protein kinase 10).

## 4 Discussion

Flavonoids (flavones, flavan-3-ols, isoflavones, flavanones, flavonols, and anthocyanins) contribute to tea taste: the bitterness and astringency in tea infusion (He et al., 2020). In this study, we found that total flavonoid content in young leaf tended to increase with tree age. However, this indicator alone does not directly indicate tea quality because leaf processing also affects tea quality. Similarly, the phenol–amino acid ratio is seen as an important indicator of tea quality (Ku et al., 2010); a decrease in it could not signal a decline in tea quality. Amino acids contribute to tea infusion taste. Of amino acids, only theanine, glutamic acid, aspartic acid, and asparagine have a umami taste; other amino acids present bitter or sweet flavors (Zhang et al., 2020). We report the levels of theanine, glutamic acid, valine, and alanine were significantly higher in leaves of 10-y-old tea trees than in 80- and 800-y-old tea trees. Theanine, which accounts for > 50% of total amino acids in tea leaves, is a key contributor to the umami taste of tea infusion (Kaneko et al., 2006). Glutamic acid is also a umami-related amino acid (Zhou et al., 2020). This suggests that the umami taste in tea infusions from older trees would be weaker. However, the taste of tea infusion is formed from the synthetic perception of various taste-active compounds, with different compounds having different contributions to specific tastes.

Sugars (e.g., fructose and galactose) and organic acids (e.g., trans-citridic and L-citramalic acids) are classic sweetness and sourness-related compounds, respectively (Hufnagel and Hofmann, 2008; Yin et al., 2022). An increase in these metabolites should improve the acidity and sweetness in tea from older trees. Similar results were reported by Chen et al. (2024), who reported the quality of black tea from old (60 y) trees to be sweeter and sourer than tea from young (5 y) trees using e-tongue technology and sensory evaluation. Lipids are important components that influence tea quality. Lipid oxidation and degradation influence aroma formation (such as heptanal and jasmine), which contribute to tea flavor (Chen et al., 2019; Zeng et al., 2018). However, how 1-alpha-linolenoyl-glycerol and 2-aphla-linolenoyl-glycerol affect tea quality remains unknown.

Phenolic acids, flavonols (flavones), catechins, and their oxidation products are important taste contributors in black tea. Phenolic acids (such as coumaroylquinic and caffeoylquinic acids) are taste compounds or modifiers (Cao et al., 2019; Kaneko et al., 2006) and bitter and astringent components in black tea (Wen et al., 2022). Flavonols (such as quercetin 3-O-rutinoside) correlate positively with bitter and astringent tastes (Chen et al., 2022). Catechins (EGCG, EGC, and GCG) also have astringent and bitter tastes in black tea (Xu et al., 2018). The higher the content of catechins (e.g., theaflavin and theaflavin-3-O-gallate), the deeper the tea infusion color (Dai et al., 2017). We also found lower phenolic acid and flavonol (flavone) contents and higher content of catechins in leaves of older trees, possibly resulting in less bitter and astringent taste and darker color of the tea infusion.

Long et al. (2024) reported the quality of three types of Yunnan Congou black teas from trees of different ages (decades, hundreds, and a thousand years). They reported that with increased tree age, the umami and sweetness scores of tea infusion increased significantly, but astringency and bitterness decreased. Infusion from thousand-year-old trees was significantly less bitter and astringent, and had sweet and umami characteristics, producing a sweet-mellow sensation. In addition, Chen et al. (2024) reported black tea produced from new leaves of 60-y-old trees to be stronger and sweeter, sourer, and richer, and less bitter and astringent than tea from 5-y-old trees. We also performed a comparative sensory evaluation of black tea samples from young and old *C. tachangensis* var. remotiserrata. plants based on prior research (Wang, 2009) (**Supplementary Table S4**, **Supplementary Figure S7**). The result indicated that black tea produced from old trees was characterized by a more pronounced sweetness and richness, as well as weaker bitterness and astringency. Although it is established that raw material dictates final tea quality under standardized processing, our findings specifically demonstrate that plant age modulates the metabolism of substances such as sugars, amino acids, phenolic acids, and flavonoids. The consistency between these metabolic shifts and the sensory profile of the tea substantiates the role of plant age in determining quality. It should be noted that this conclusion is based on analysis from the spring harvest of 2023 alone. Therefore, validating the mechanism by which plant age affects quality via leaf metabolism necessitates future studies incorporating more diverse sampling across multiple growing seasons and geographical locations.

Phenylpropanoid metabolism is an important metabolism in plants, yielding many metabolites that contribute to plant development and plant–environment interplay. In our study, we found phenylpropanoid metabolic pathways differ between young and old tea trees, indicating the adaptive strategy of woody perennial plants is age-related. The result is consistent with the reprots of Chang et al. (2019) and Wang et al. (2020) in which they used *Platycladus orientalis* and *Ginkgo biloba* as materials, respectively. Regulation of phenolic acid and flavonoid biosynthesis involves transcriptional and post-transcriptional mechanisms. Throughout these processes, TFs play a pivotal role, with the MYB-mediated control of flavonoid biosynthesis serving as the most paradigmatic example. The MYB family of TFs often act in complex with bHLH and WD40 repeat proteins to form the MBW complex. This complex activates the expression of structural genes involved in flavonoid synthesis (Shen et al., 2022). Zhao et al. (2023) reported that while the CsMYB75- or CsMYB86-directed MYB-bHLH-WD40 (MBW) complexes differentially activate anthocyanin or catechin biosynthesis in tea leaves, respectively, CsMYBL2a and CsMYBL2b homologs negatively modify the light- and temperature-induced anthocyanin and catechin production in both Arabidopsis and tea plants. Other TF families (bZIP, WRKY, and MADS-box) also play roles in regulating flavonoid biosynthesis in response to environmental stimuli and developmental cues (Naik et al., 2022).

With improved knowledge of transcriptional and post-transcriptional regulation of phenylpropanoid metabolism, the role of epigenetic regulation in the control of phenylpropanoid metabolism is being increasingly investigated. With increased age, perennial plants experience more abiotic and biotic stresses that are closely related to epigenetic regulation of gene expression, explaining why age leads to differences in metabolic pathways. In *Malus crabapple*, downregulation of histone deacetylase 6 correlates with reduced transcription of methyltransferase 1 and promotes McMYB10 expression and anthocyanin content (Peng et al., 2020). Spreading of DNA methylation is also involved in epigenetic regulation of phenylpropanoid metabolism. In red-fleshed radish (*Raphanus sativus* L.), the hypermethylated CACTA transposon upstream of the RsMYB1 promoter induces the spread of DNA methylation to the promoter of RsMYB1 and represses its expression; as a consequence, anthocyanin content decreases (Wang et al., 2020). We also report H3K4 and MET1 to be DEGs with increased tree age (**Supplementary Figure S2**). Therefore, elucidating the influence of tree age on the methylation status of key genes—particularly the aforementioned TFs—in tea plants represents a pivotal direction for future research.

Among the genes exhibiting up-regulated expression with increasing tree age, we identified several enzymes and TFs. Phenylalanine ammonia-lyase and GDSL esterase/lipase are closely related to amino acid and lipid metabolism. The expression of genes PAL and GDSL increases with age, explaining changes in amino acids and lipids. TFs such as SAUR50 and GRF1 may be directly related to regulation of plant secondary metabolism. It has been reported that GRF1 participates in regulation of defense-related TFs, cell-wall modification, cytokinin biosynthesis and signaling, and secondary metabolite accumulation (Wang et al., 2021); the SAUR gene might be involved in metabolic regulation of plant growth and development, and protection against stressors (Yan et al., 2024). Whether these TFs function in specific secondary metabolic pathway changes led by age requires further investigation.

## 5 Conclusion

**Figure 8:**
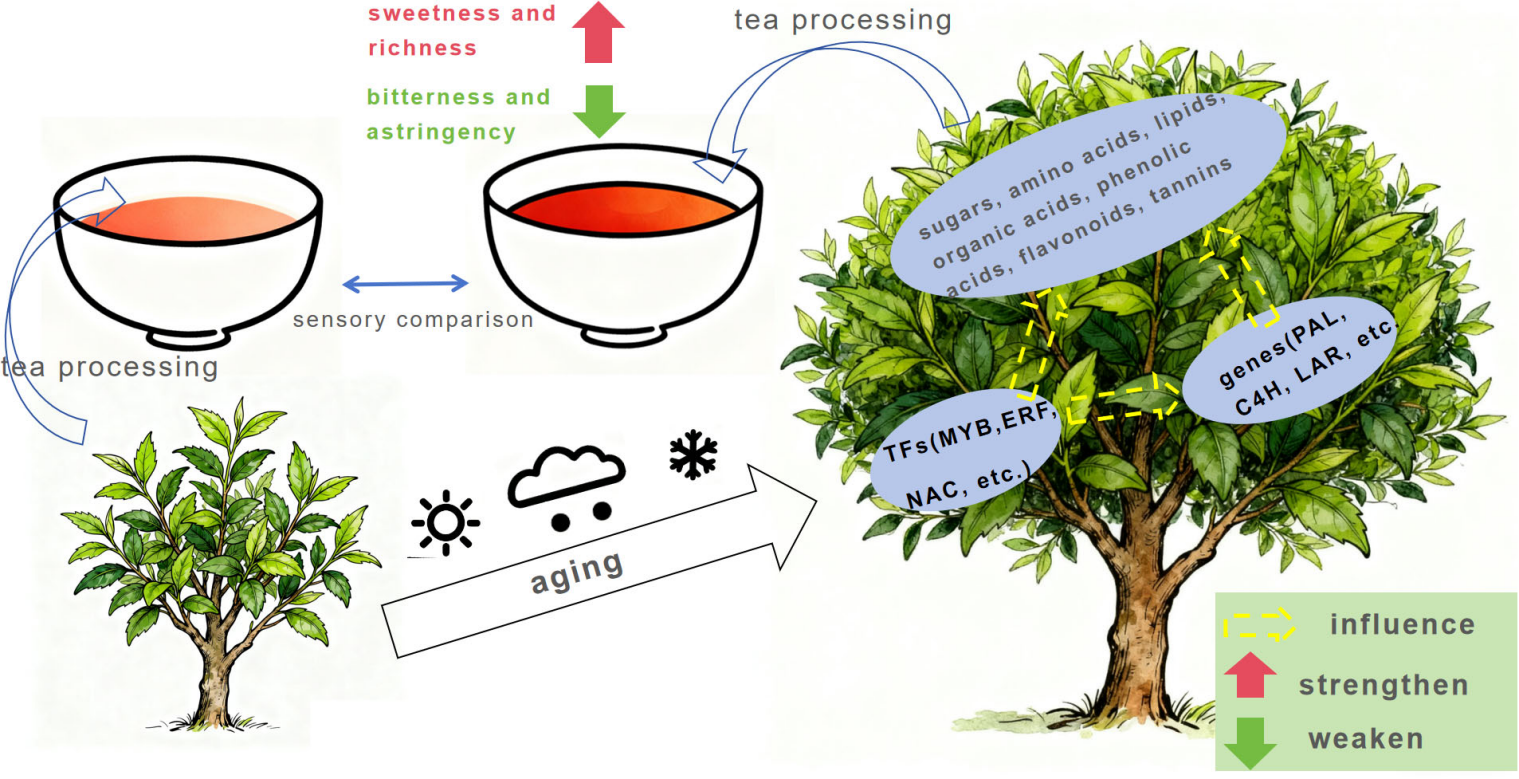
The proposed mechanism for the impact of tree age on tea quality.

We systematically investigate the impact of tree age on metabolism of tea quality-related compounds using metabolomics and transcriptomics. We report tree age to affect primary and secondary metabolic processes in new tea leaves in spring, including an increase in specific sugars and lipids, a decrease in specific amino acids, phenolic acids, and flavonoids, and accumulation of catechins. This is consistent with corresponding changes in taste, aroma, and tea infusion color from older trees (increased sweetness, decreased bitterness and astringency, enhanced aroma, and deeper color). Tree age also modulates expression of specific genes in new tea leaves, thereby regulating the metabolic pathways of quality-related compounds, particularly genes involved in the phenylpropanoid metabolic pathway (e.g., flavanone 3β-hydroxylase gene, anthocyanidin reductase gene). Expression profiles of many MYB, WRKY, NAC and ERF TF genes are also closely related to changes in compounds such as phenolic acids and flavonoids, implying their important regulatory roles in secondary metabolic processes of tea trees. Based on this, a plausible mechanism for the influence of tree age on tea quality can be postulated, which is depicted in **Figure 8**. It should be noted that the predicted functions of these TFs await further experimental validation.

## Data Availability

The transcriptomic and metabolomic data presented in this study can be found in the online repository: China National Center for Bioinformation (https://ngdc.cncb.ac.cn, BioProject No. PRJCA049217).
