# Supplementary material for: Determining the influence of tree age on tea quality-related metabolites in Camellia tachangensis var. remotiserrata by integrating metabolomic and transcriptomic analyses
Source: Front Plant Sci. 2025 Nov 10;16:1701011. doi: 10.3389/fpls.2025.1701011 (PMC12640977; doi:10.3389/fpls.2025.1701011)
Supplement: Supplementary file 1 [file DataSheet1.docx]

**Supplementary data**

**Determining the influence of tree age on tea quality-related metabolites in *Camellia tachangensis* var. remotiserrata by integrating metabolomic and transcriptomic analyses**

Zhuorong Shi^1^, Yu Cai^2^, Ying Tian^3^, Yuting Zhang^1^, Junhao Shao^1^, Jianfeng Liu^1^, Qingwei Zhang^1^*

^1^Key Laboratory of Eco-Environments of Three Gorges Reservoir Region, Ministry of Education, Southwest University, Tiansheng Road No. 2, Beibei, Chongqing 400715, China

^2^Chongqing Haotian Eco-Agri Tech Co., Ltd., Minsheng Road No. 69, Shiqiang, Nanchuan, Chongqing, 406407, China

^3^Bureau of Agriculture and Rural Affairs of Langzhong City, Chang’anxi Road No. 219, Langzhong, Nanchong, Sichuan, 637455, China

*Corresponding author at: Southwest University, Tiansheng Road No. 2, Beibei, Chongqing 400715, China. E-mail address: qwzhang18@swu.edu.cn

**Table S1** The growth characteristics of tea tree in the study

| Tree age | Sample | Diameter at breast height (cm) | Crown width（cm） | Tree height (m) |
| --- | --- | --- | --- | --- |
| 10 | Y | 7.01±1.12 c | 75.0±10.6 c | 2.63±0.68 c |
| 80 | H | 18.09±1.75 b | 290.8±23.5 b | 5.11±0.87 b |
| 800 | T | 29.42±2.25 a | 387.3±46.3 a | 7.77±1.12 a |

Y: 10-year old; H: 80-year old; T: 800-year old. The data are mean value ± SD (n=3); different letters indicate there is significant difference (ANOVA, P＜0.05)

**Table S2** Sequencing output statistics of transcriptome analysis

| Sample | Raw Reads | Clean Reads | Clean Base(G) | Mapped Reads/Clean Reads | Unique mapped Reads/Clean Reads |
| --- | --- | --- | --- | --- | --- |
| Y1 | 43896014 | 42105804 | 6.32 | 85.34% | 74.62% |
| Y2 | 46307594 | 44766808 | 6.72 | 85.94% | 74.06% |
| Y3 | 44738718 | 43062354 | 6.46 | 84.37% | 73.31% |
| H1 | 48596702 | 46868248 | 7.03 | 81.83% | 71.00% |
| H2 | 50459644 | 48672186 | 7.30 | 86.29% | 74.16% |
| H3 | 49236584 | 47658688 | 7.15 | 85.19% | 73.42% |
| T1 | 48170124 | 46508190 | 6.98 | 86.89% | 74.84% |
| T2 | 48230324 | 46219356 | 6.93 | 86.23% | 74.53% |
| T3 | 44279138 | 43053724 | 6.46 | 85.94% | 74.61% |

Y: 10-year old; H: 80-year old; T: 800-year old.

**Table S3** Genes and corresponding primers used for quantitative expression analysis**.**

| **No.** | **ID** | **Gene** | **Full name** | **Forward primer sequence(5'-3')** | **Reverse primer sequence(5'-3')** |
| --- | --- | --- | --- | --- | --- |
| 1 | CSS0016393 | AAP3 | Amino acid permease 3 | ATGCTCTGTGGCTGCT | AGGAGCCAGTAGGGAT |
| 2 | CSS0004403 | C4H | cinnamate 4-hydroxylase | CGAAGATGGTCCGCAC | CCAGCCTCTTGTTGTAC |
| 3 | CSS0015062 | CD48C | Cell division control protein 48 homolog C | AGATGCCAGTGTGGAT | CTTCATTCATCAGTGCAG |
| 4 | CSS0028360 | CESA2 | Cellulose synthase A catalytic subunit 2 [UDP-forming] | AGACGACTGGTGGAGA | TGGAGGTAACAGTGAAG |
| 5 | CSS0012595 | CHI | chalcone isomerase | TGCTGTGGATGCCAC | AGACACTGGATCACTTC |
| 6 | CSS0004474 | CHS1 | Chalcone synthase 1 | ATAGTGACGGTGCCA | CCCAGGAACATCCTTG |
| 7 | CSS0046428 | CML2 | Calmodulin-like protein 2 | TCATCACGGTGGAGGA | ATGATCATCCTCCGGC |
| 8 | CSS0031491 | CYP72A219 | Cytochrome P450 CYP72A219 | AGCCCTAGCAATGATT | CGTGCACGTATGATGG |
| 9 | CSS0034413 | ERF3 | Ethylene-responsive transcription factor 3 | GTCGTCTGTGATCGAC | TCGACGTCTTCAAGAG |
| 10 | CSS0044072 | EXPA8 | Expansin-A8 | ACTTCAACCTAGTGTTG | AGTTCCTTGACATGG |
| 11 | CSS0047476 | F3GT2 | Flavonol 3-O-glucosyltransferase | GCACATCCATCGGTTG | GCATCCTGTACCATACTA |
| 12 | CSS0002446 | FAD3C | Omega-3 fatty acid desaturase | CTGCTAGGCCTGTGCT | CAGAGAGCTGAGGATCTG |
| 13 | CSS0007984 | FAOMT | Flavonoid 3',5'-methyltransferase | CGTGGTTCAGTTGTAAGC | GCTAAGTGCTTGTTGAGT |
| 14 | CSS0003382 | FL3H2 | Flavanone 3-beta-hydroxylase 2 | AGCTCAGTTGAACGAACA | CGCTCGAGCTCATCTT |
| 15 | CSS0013849 | GDSL32 | GDSL esterase/lipase 32 | GTGGTAGTGGTATCACCT | CATCCTGTCCGCAACATG |
| 16 | CSS0015908 | H3K4 | COMPASS-like H3K4 histone methylase component WDR5B | ACATCGTCAGCGGATC | ATCAGAGTGTCCGTCA |
| 17 | CSS0007135 | LAC14 | Laccase-14 | TAGAGCTGATAATCCAGG | CAATTAGCACTGTATCCA |
| 18 | CSS0034690 | LAR | Leucoanthocyanidin reductase | ATCCAGATGCATCATTTC | ACTGAAGCAACACTAATC |
| 19 | CSS0042457 | MET1-1 | DNA (cytosine-5)-methyltransferase 1A | AGACACAATCAATGGAAG | GAGTATCCTGTCCTGGT |
| 20 | CSS0014929 | METK2 | S-adenosylmethionine synthase 2 | GGTGCATTGTGCAGGT | GCAATCATTCCAGGCCTA |
| 21 | CSS0028845 | MY123 | Transcription factor MYB123 | AATGAGATGCTCCAAGAT | AGCTCTGCTAATAATGGC |
| 22 | CSS0022757 | MYB308 | Myb-related protein 308 | CTGATGCTGGAAGTTGT | GATATGGTGGACTCATCA |
| 23 | CSS0031776 | MYB86 | Transcription factor MYB86 | ATGTCAATAGCCACACTG | CATGGACTCGGCTTCA |
| 24 | CSS0029111 | MYC2 | Transcription factor MYC2 | ACCACCCTGCAGCTAG | GTTCACCACGGAGACAC |
| 25 | CSS0048281 | PALY | Phenylalanine ammonia-lyase | CCAGGATGTGAACTCTCT | CTCCTCCAAATGCCTCAA |
| 26 | CSS0001194 | PER4 | Peroxidase 4 | CAGGCACCTTCAACTCT | CTCCATTCGATCCAGTGA |
| 27 | CSS0008291 | PILS5 | Protein PIN-LIKES 5 | TGATTGTTGCTGTAATCT | AGCGGTACAACGGATC |
| 28 | CSS0023866 | PPAF2 | Purple acid phosphatase 2 | AGCATACATCATCGTTCT | AGAACAATCAACCATGGC |
| 29 | CSS0040677 | SAU50 | Auxin-responsive protein SAUR50 | ATCTACGACGACGACAAC | GGAACTCAGGGTGAGTCA |
| 30 | novel.15096 | SSY2 | Granule-bound starch synthase 2 | TTAGGTTACTGTTGAGTT | GAGTAAGCATGTTATGCA |
| 31 | CSS0004506 | U88F5 | UDP-glycosyltransferase 88F5 | ATGGTCGCCGGAGTTC | TTCATATCCTCCACCATC |
| 32 | CSS0033291 | UGPA | UTP--glucose-1-phosphate uridylyltransferase | ACCTTGAATGATGGCTAT | ACTTGAAACGACTCAAGA |
| 33 | CSS0028565 | WRK40 | WRKY transcription factor 40 | GGTAGCAACATATGAAGG | TAGTGTCATGCCTCGGT |
| 34 | CSS0000110 | UBQ | Ubiquitin domain-containing protein DSK2a | AGGGTAGCACTAGAACAC | AGCCACCAGCTCCGAG |

**Table S4** Sensory evaluation of tea produced by tea trees of different ages

| **Evaluation Indicator** | **10-year-old Tea Trees** | **100-year-old Tea Trees** |
| --- | --- | --- |
| Appearance | The tea leaves are plump, tight and well-formed, with a dark green and glossy color. | The tea leaves are plump, tight and well-formed, with a dark green and glossy color. |
| Infusion Color | Dark red, thick and bright | Dark red, thick and bright, with a distinct golden rim |
| Aroma | Rich sweet aroma, accompanied by the fragrance of fine hairs | Rich, mellow and long-lasting sweet aroma |
| Taste | Mellow and thick | Fresh, strong and with a sweet aftertaste |
| Infused Leaves | Red, uniform and bright | Red, soft, uniform and bright |

Note: The Sensory Evaluation was conducted according to the method established by Zeng et al. (Zeng L., ZHang B., Wei F., Luo L. 2023.Optimization of QDA Analysis Conditions and Flavor Wheel Establishment for *Camellia nanchuanica* Black Tea. Journal of Tea Communication, 50(2): 141-152.)


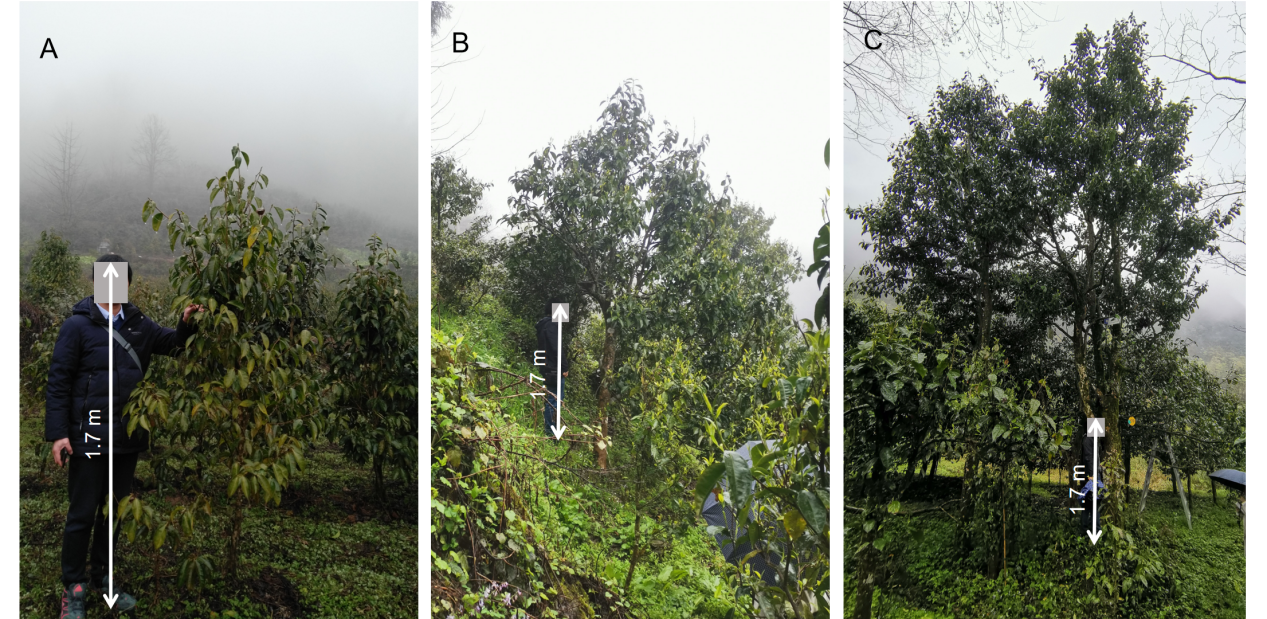


Fig. S1 The growth status of tea tree in the study. A:10-year old; B: 80-year old; C: 800-year old.


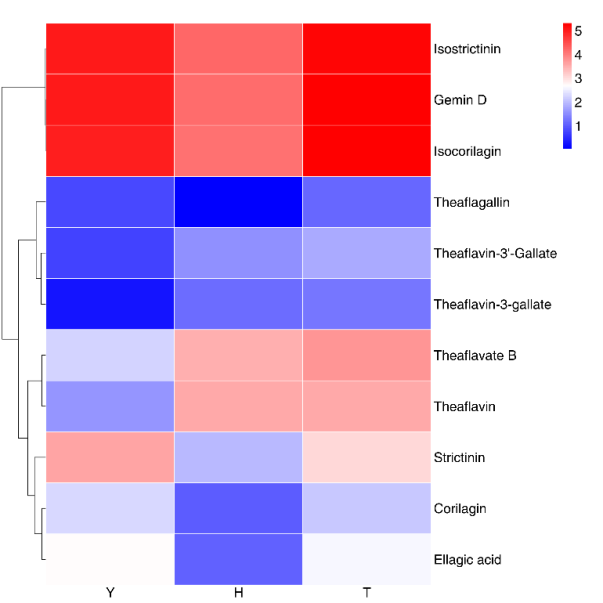

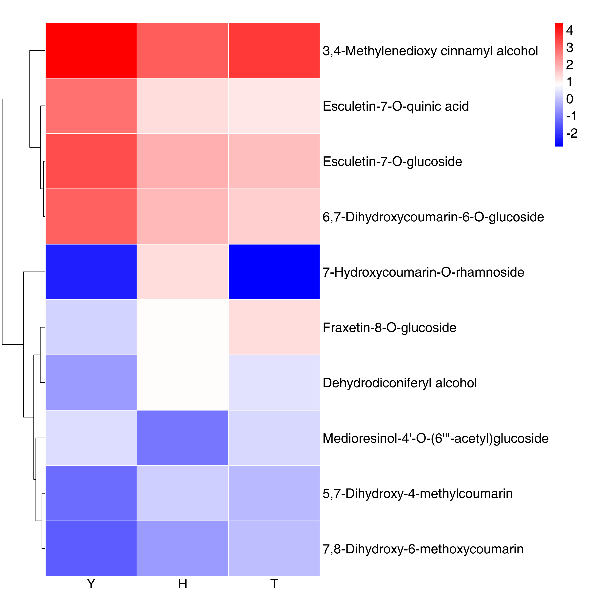


Fig. S2 The changes of tannins, lignans and coumarins in young leaves of tea plants with various ages. Y: 10-year old. H: 80-year old. T: 800-year old.


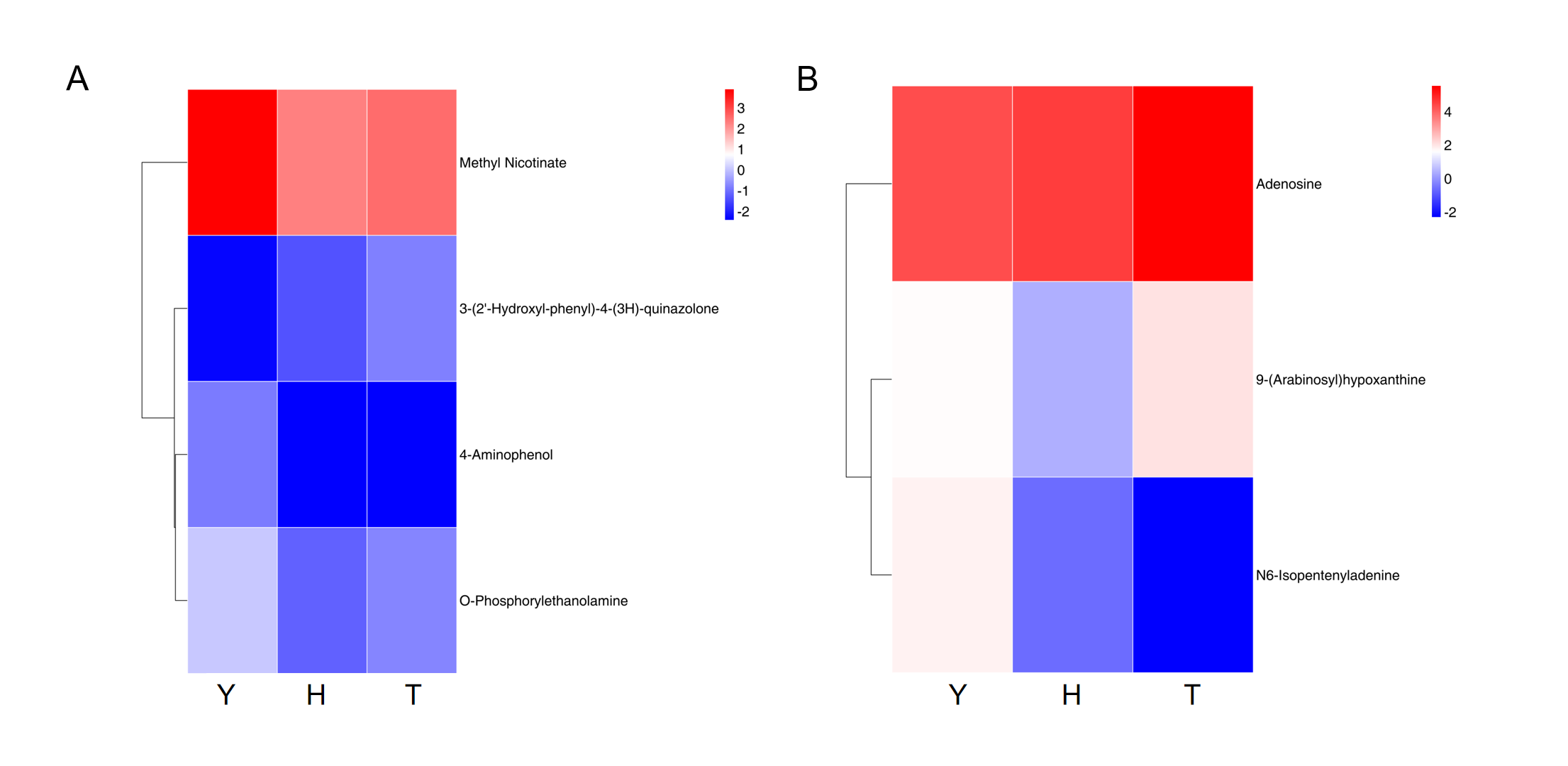


Fig. S3 The changes of alkaloids (A) and nucleosides and their derivatives (B) in young leaves of tea plants with various ages. Y: 10-year old. H: 80-year old. T: 800-year old.


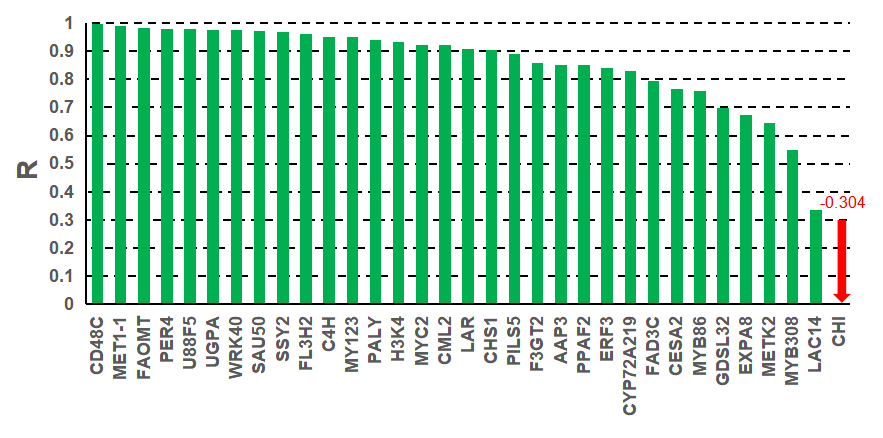


Fig. S4 Correlation coefficient (R) between gene expression levels and FPKM values.


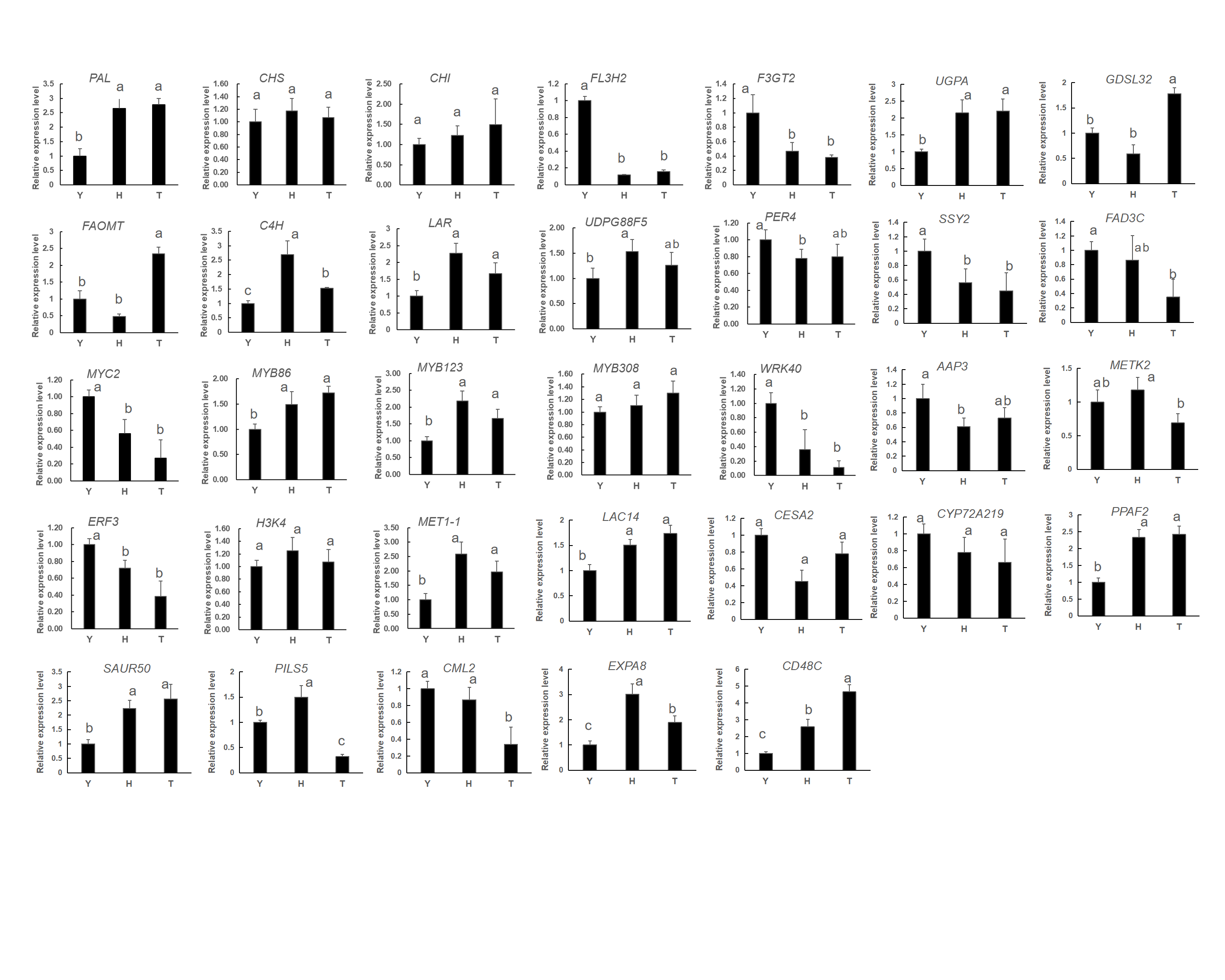


Fig. S5. The relative expression levels of selected genes in yong leaves in tea trees with different ages. Y: 10-year old; H: 80-year old; T: 800-year old. Different lowercase letters (a, b, c) indicate a significant difference (ANOVA, P < 0.05). AAP3:Amino acid permease 3; C4H: cinnamate 4-hydroxylase; CD48C: Cell division control protein 48 homolog C; CESA2: Cellulose synthase A catalytic subunit 2 [UDP-forming]; CHI: chalcone isomerase ; CHS1: Chalcone synthase 1; CML2:Calmodulin-like protein 2; CYP72A219: Cytochrome P450 CYP72A219; ERF3: Ethylene-responsive transcription factor 3; EXPA8: Expansin-A8; F3GT2:Flavonol 3-O-glucosyltransferase; FAD3C: Omega-3 fatty acid desaturase; FAOMT: Flavonoid 3',5'-methyltransferase; FL3H2: Flavanone 3-beta-hydroxylase 2; GDSL32: GDSL esterase/lipase 32; H3K4: COMPASS-like H3K4 histone methylase component WDR5B; LAC14: Laccase-14; LAR:Leucoanthocyanidin reductase; MET1-1: DNA (cytosine-5)-methyltransferase 1A; METK2: S-adenosylmethionine synthase 2; MY123: Transcription factor MYB123; MYB308: Myb-related protein 308; MYB86: Transcription factor MYB86;

MYC2: Transcription factor MYC2; PALY: Phenylalanine ammonia-lyase; PER4:Peroxidase 4; PILS5: Protein PIN-LIKES 5; PPAF2: Purple acid phosphatase 2; SAU50: Auxin-responsive protein SAUR50; SSY2: Granule-bound starch synthase 2; U88F5: UDP-glycosyltransferase 88F5; UGPA:UTP--glucose-1-phosphate uridylyltransferase; WRK40: WRKY transcription factor 40.


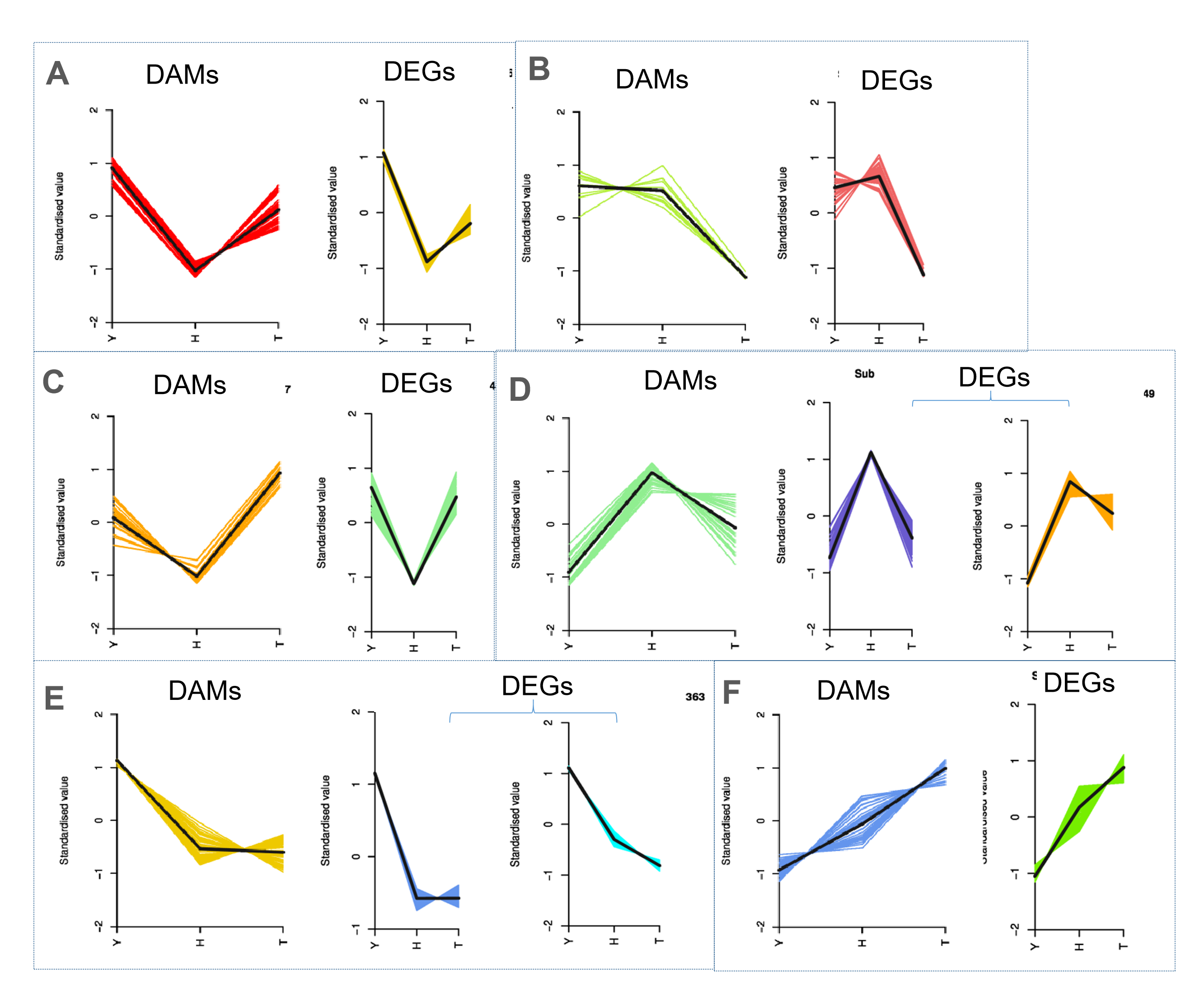


Fig. S6.The integrated clustering analysis of differentially accumulated metabolites (DAMs) and differentially expressed genes (DEGs). Y: 10-year old; H: 80-year old; T: 800-year old.


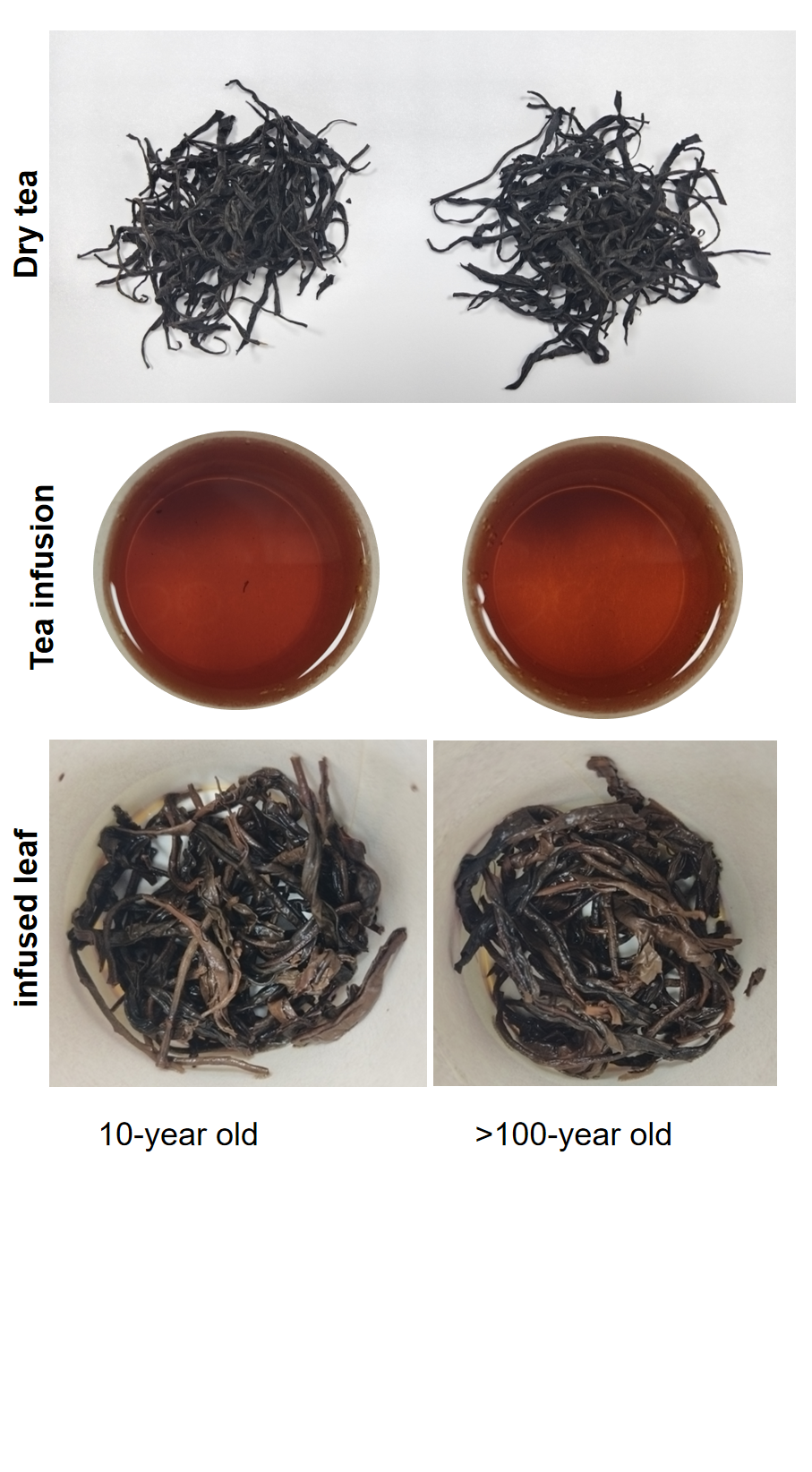


Fig. S7 Dry tea, tea infusion and infused leaf of black tea harvested from young tea tree and old tea tree.
